# Prognostic value of blood lactate and glucose levels after aneurysmal subarachnoid hemorrhage

**DOI:** 10.1186/cc14546

**Published:** 2015-03-16

**Authors:** S Dijkland, K Van Donkelaar, W Van den Bergh, J Bakker, D Dippel, M Nijsten, M Van der Jagt

**Affiliations:** 1 Erasmus MC - University Medical Center, Rotterdam, the Netherlands; 2 University Medical Center Groningen, the Netherlands

## Introduction

In critically ill patients, blood lactate on admission is associated with outcome, but in patients with aneurysmal sub-arachnoid hemorrhage (SAH) this has not been investigated. We studied the association of early circulating lactate and glucose with unfavorable disease course. The prognostic role of both lactate and glucose was studied, hypothesizing that both may be increased due to sympathetic activation after SAH [1].

## Methods

In this retrospective cohort study we included consecutive patients with aneurysmal SAH admitted to the ICUs of two university hospitals in the Netherlands between November 2006 and December 2011. Exclusion criteria were: nonaneurysmal SAH, ICU admission >24 hours after ictus, death ≤48 hours after admission and no lactate measurement <24 hours after admission. Maximum blood lactate and glucose levels within the first 24 hours after SAH were used for analyses. The outcomes were DCI, defined as a new hypodensity on brain CT due to DCI, and poor outcome, defined as a modified Rankin Scale of 4, 5 or death 3 to 6 months after the ictus. We performed proportional hazard analyses to assess the associations of lactate and glucose with DCI, and logistic regression was used to assess the associations with poor outcome. Multivariable analyses were adjusted for established predictors for DCI and poor outcome.

## Results

Two hundred and eighty-five patients were included in the analyses. DCI occurred in 84 patients (29%) and 106 patients (39%) had poor outcome. Lactate was independently associated with DCI (adjusted HR = 1.16, 95% CI = 1.04 to 1.30) and poor outcome (adjusted OR = 1.53, 95% CI = 1.25 to 1.94). Maximum lactate and glucose were strongly related (Spearman's ρ = 0.55, *P *< 0.001). In multivariable analyses including both lactate and glucose as independent variables, only lactate was independently related to poor outcome (OR = 1.42, 95% CI = 1.11 to 1.81), and only glucose was independently associated with DCI (HR = 1.10, 95% CI = 1.02 to 1.19).

## Conclusion

Maximum lactate in the acute phase after aneurysmal SAH is associated with both DCI-related infarction and poor outcome. Once glucose was considered, early lactate remained independently associated with poor outcome, while glucose, instead of lactate, was associated with DCI. These routinely available laboratory measurements may improve identification of patients at risk for complications or poor outcome after SAH. Confirmation of the pathophysiological significance of lactate and glucose in prospective research is warranted.
